# Photosynthesis Performance and Antioxidative Enzymes Response of *Melia azedarach* and *Ligustrum lucidum* Plants Under Pb–Zn Mine Tailing Conditions

**DOI:** 10.3389/fpls.2020.571157

**Published:** 2020-09-15

**Authors:** XinHao Huang, Fan Zhu, ZhiXiang He, XiaoYong Chen, GuangJun Wang, MengShan Liu, HongYang Xu

**Affiliations:** ^1^College of Life Science and Technology, Central South University of Forestry and Technology, Changsha, China; ^2^National Engineering Laboratory for Applied Forest Ecological Technology in Southern China, Central South University of Forestry and Technology, Changsha, China; ^3^College of Arts and Sciences, Governors State University, University Park, IL, United States

**Keywords:** antioxidative enzymes, *Ligustrum lucidum*, *Melia azedarach*, Pb–Zn tailings, photosynthesis

## Abstract

Lead–zinc (Pb–Zn) mine tailings pose a great risk to the natural environment and human health because of their high toxicity. In this study, the responses of photosynthesis, chlorophyll fluorescence, and antioxidative enzyme of *Melia azedarach* and *Ligustrum lucidum* in the soil contaminated by Pb–Zn mine tailings were investigated. Results showed that Pb–Zn mine tailings significantly reduced net photosynthetic rates and leaf photosynthetic pigment content of both trees, and the reduction of net photosynthetic rates was mainly caused by their biochemical limitation (BL). The chlorophyll fluorescence parameters from Pb–Zn tailing stressed leaves indicated that Pb–Zn tailings affected PSII activity which was evident from the change values of energy fluxes per reaction center (RC): probability that an electron moves further than Q_A_^−^ (ET_O_/TR_O_), maximum quantum yield for primary photochemistry (TR_O_/ABS), the density of PSII RC per excited cross-section (RC/CS_O_), the absorption of antenna chlorophylls per PSII RC (ABS/RC), and the turnover number of Q_A_ reduction events (N). Pb–Zn mine tailings also affected the oxidation and reduction of PSI, which resulted in a great increase of reactive oxygen species (ROS) contents and then stimulated the rate of lipid peroxidation. Both trees exhibited certain antioxidative defense mechanisms as elevated superoxide dismutase (SOD), peroxidase (POD), and catalase (CAT) activities, then declined under high level of Pb–Zn tailing treatment. Comparatively, *L. lucidum* showed less extent effect on photosynthesis and higher antioxidative enzyme activities than *M. azedarach*; thus *L. lucidum* was more tolerant than *M. azedarach* at least under the described Pb–Zn tailing treatment. These results indicate that the effect of Pb–Zn mine tailings on photosynthesis performance mainly related to imbalance of the PSII activity and PSI redox state in both trees. We propose that *M. azedarach* and *L. lucidum* could relieve the oxidative stress for phytoremediation under the appropriate Pb–Zn mine tailing content.

## Introduction

Plants require certain heavy metals (HMs) as essential elements for their growth, development, and yield production, but excess amount of these metals can become phytotoxic and cause adverse effects on plant biomass production, crop yield, and food safety (Pierattini et al., 2017). The major sources of HMs originated from anthropogenic activities; mining activities seem to be the largest contributor of HM pollutions in many places, and it is a particular case in China (Ha et al., 2011; Han et al., 2013; Zhuang et al., 2014; Chen et al., 2015). It was recently reported that the mining activities have resulted in about 40,000 ha mining wastelands in this country, and the wastelands have been continuously expanded at a rate of 3,300 ha per year (Li, 2006; Luo et al., 2015; Yu et al., 2019). Mining activities generated a large amount of mine tailings in the mining sites where high concentrations of Pb, Zn, and other HMs were detected in local environments, which caused a wide range of environmental problems (Han et al., 2013). Therefore, it is urgent and necessary to reestablish vegetation in mining wasteland for ecological restoration (Yu et al., 2019).

Various methods have been identified and employed to the restoration of HM-polluted soils (Teng et al., 2015). Phytoremediation technology has been widely considered as an efficient, inexpensive, and environmental-friendly approach to clean up the contaminated environments by HMs (Han et al., 2016). Trees have the great potential use in remediation of HM-polluted soil in view of large biomass and massive root systems (Pulford and Watson, 2003). However, Tang et al. (2019) demonstrated that the Pb–Zn mine tailing significantly reduced plant growth and chlorophyll contents in three woody plants (*Castanopsis fissa*, *Daphniphyllum calycinum* and *Pinus massoniana*). The limitation in tree growth might thus be associated with photosynthesis which was sensitive to HM stress (Çiçek et al., 2017; Zhong et al., 2018; Huang et al., 2019; Liang et al., 2019). Evidence showed that the reduction of leaf photosynthesis under HM toxicity might be attributed to the limitation of stomatal opening (Gs) and CO_2_ diffusion in mesophyll (Gm), the suppression of photochemistry and biochemistry, or synthetic combinations of these factors (Kosobrukhov et al., 2004; Kola and Wilkinson, 2005; Krantev et al., 2008; Deng et al., 2014). Sagardoy et al. (2010) reported the main limitation to photosynthesis rate in sugar beet was likely due to the reduction of Gs under Zn stress. Lin and Jin (2018) found similar results in an experiment with three vegetables (*B. chinensis*, *C. coronarium*, and *B. alboglabra*) under Cu stress. However, Velikova et al. (2011) studied the impact of Ni on the photosynthesis of *Populus nigra* and found the limitation to photosynthesis rates of this species resulted from the restriction in Gm, not in Gs. Gm influenced photosynthetic capacity and determined the available CO_2_ concentration for photosynthesis in the chloroplasts (Centritto et al., 2009). Some studies showed that the electron transfer was inhibited by Pb pollutant at the photosystem I (PSI) donor side because Pb affected activity of PSI (Belatik et al., 2013; Bernardini et al., 2016), but some studies suggested the activity of PSI in *Microcystis aeruginosa* and *Chlorella pyrenoidosa* had no inhibition with Cd treatment (Zhou et al., 2006; Wang et al., 2013). And some studies indicated that Cd affected the whole electron transport in photosystem II (PSII): on the donor side, it inhibited the OEC, while on the acceptor side, it inhibited electron transport between Q_A_^−^ and Q_B_^−^. (Mallick and Mohn, 2003; Sigfridsson et al., 2004; Chu et al., 2018). Additionally, a number of studies also reported that HMs exhibited less effect of photosynthetic rate, electron transport, conversion of light energy, and photochemical efficiency in tolerant plant species than those in sensitive ones (Guo et al., 2018; Sorrentino et al., 2018; Huang et al., 2019).

Reactive oxygen species (ROS) can highly increase in the chloroplasts when photosynthetic electron transport chain was blocked by HM toxicity (Zhang et al., 2018). ROS like superoxide (O_2_^−^) and hydrogen peroxide (H_2_O_2_) could lead to lipid peroxidation, protein oxidation, membrane and nucleic acid damage, and inactivation of enzymes (Bi et al., 2016; Bezerril et al., 2017; Lu et al., 2017). To prevent an oxidative damage, plants activated enzymatic ROS scavenging mechanisms, such as superoxide dismutase (SOD), peroxidase (POD) and catalase (CAT), ascorbic acid and glutathione, to keep ROS at a basal non-toxic level (Israr et al., 2011; Štefanić et al., 2018; Du et al., 2020). In the past two decades, many studies have estimated the direct link between the oxidative stress in metal toxicity and metal-tolerant plants (Boominathan and Doran, 2003; Chiang et al., 2006). A large proportion of studies have indicated that metal tolerant plants were linked to superior constitutive antioxidative defense (Srivastava and Doran, 2005; Chiang et al., 2006; Nie et al., 2016). Although great progress has been made in supporting the HM toxicity to plant photosynthesis and its redox balance, the effects of HM stress on photosynthetic performance varied depending on the plant species, metal ion and concentration. Meanwhile, these available data provide a restricted view on single metal contamination or herbaceous plants (Mobin and Khan, 2007; Wali et al., 2016). Therefore, the photosynthesis and redox responses of trees to HM tailing stress still need to be studied further.

*Melia azedarach* is a fast-growing, deciduous broad-leaved tree species and is widely distributed in the southern regions of China. It is mainly planted for reforestation as a useful timber production species or as an ornamental plant. *Ligustrum lucidum* is a commonly-seen evergreen broad-leaved tree species in South China. This species is often planted as an ornamental tree in the urban. Moreover, both *M. azedarach* and *L. lucidum* are native plants in the southern regions of China, Frérot et al. (2006) pointed out that native plants could be a useful option to phytoremediation because native plants are better adapted to local climate conditions than plants introduced from other environments, and they were previously found to have high tolerance in Cd or Mn contaminated soils (Trikshiqi and Rexha, 2015; Su et al., 2017; Liang et al., 2019). However, the metal toxicity manifestations and mechanisms behind tolerance need further investigation, so the specific goals of this study were: 1) to investigate the contributions of Gs, Gm, and biochemical component respectively to the photosynthesis reduction in two tested plants by photosynthesis limitation analysis, 2) to identify the impact of Pb–Zn stress on the whole photosynthetic electron flow chain from PSII to PSI and the state of PSI, 3) to explore the tolerant mechanisms of both trees to Pb–Zn tailing stress in terms of the redox responses of photosystems and antioxidative enzymes. Based on the above studies, the differences of tolerance between *M. azedarach* and *L. lucidum* were discussed. Results could provide a theoretical basis for selecting and breeding the resistant trees species grown in Pb–Zn polluted environments.

## Materials and Methods

### The Physicochemical Properties of Pb–Zn Tailings and Experimental Soil

The study was conducted in the Central South University of Forestry and Technology (CSUFT), Changsha City, Hunan Province, China (28°8′12″N, 112°59′36″E). The Pb–Zn mine tailing samples were collected from a Pb–Zn mining site in Suxian district, Chenzhou City, Hunan Province, China (25°30′38″–25°00′19″N, 112°16′41″–112°53′23″E). The soil samples were collected from the top soil (5–20 cm) in a garden field of CSUFT campus. Both Pb–Zn mine tailing samples and top soil samples were air-dried at room temperature. The large debris, stones, and pebbles were manually removed before being applied to the pot experiment. The soil pH value, soil total carbon (TC), total nitrogen (TN), total phosphorus (TP), and soil heavy metal content were measured in laboratory according to Bao (2000), the basic physicochemical properties of soil were as follows: pH 4.95, total C 5.82 g kg^−1^, total N 0.33 g kg^−1^, total P 0.16 g kg^−1^, Pb 0.002 g kg^−1^, Zn 0.003 g kg^−1^. The properties of Pb–Zn mine tailing samples were determined: pH 3.89, total C 13.89 g kg^−1^, total N 1.25 g kg^−1^, P 0.82 g kg^−1^, Pb 8.92 g kg^−1^, Zn 14.41 g kg^−1^.

### Plant Seedlings

Two-year-old young plants of *M. azedarach* (mean tree height: ~73.5 cm, mean stem base diameter: ~0.6 cm) and *L. lucidum* (mean tree height: ~130.0 cm, mean stem base diameter: ~0.9 cm) were purchased from a local nursery.

### Experimental Design

Pb–Zn mine tailings represent a poor spoil substrate characterized by high contents of Pb and Zn, low levels of organic nutrients, and poor physical structure and water retention capacity (Meeinkuirt et al., 2012); few plants can survive in such harsh environmental condition. Mixing mine tailings with garden soil helps decrease the bioavailability of metals and improves mine tailing structure and ultimately upgrades the physical properties and nutrient status of mine tailings. Moreover, this method has been commonly used in many studies of phytoremediation in mine tailing (Chen et al., 2015; Han et al., 2016; Tang et al., 2019). In the present study, four treatments were set up in the pot experiment with different weighted proportions of Pb–Zn mine tailings and garden soils. Each pot contained 10 kg mixed soils. The four treatments were: (1) 90% garden soils + 10% Pb–Zn mine tailings (designed as L1, the 10 kg mixed soils were 9 kg garden soil and 1 kg mine tailings, the same as below); (2) 75% garden soils + 25% Pb–Zn mine tailings (L2); (3) 50% garden soils + 50% Pb–Zn mine tailings (L3), and (4) 100% garden soils + 0% Pb–Zn mine tailings as the control (C). The Pb–Zn mine tailings and garden soils were completely mixed and then the *M. azedarach* and *L. lucidum* young plants were transplanted into the pots with one plant per pot. Each treatment was replicated six times. The temperatures of the greenhouse were set at 30/25°C (day temperature for 10 h and night temperature for 14 h), and relative humidity was 65/85%. During the study period, each pot was supplied with equal quantity of pure water every 1 to 2 days until the young plants were harvested. Both trees were measured for all physiological parameters after growing for 425 days (from June 15^th^, 2017 to September 13^th^, 2018) in Pb–Zn tailing treatments.

### Relative Growth Rate (RGR)

Plant biomass was measured using a harvesting method. The plant was divided into leaves, stem, and root components; the fresh weights of each component were measured by using an electronic balance and then dried at 70°C until constant weight was reached. The dry weight (DW) of each component was determined by using an electronic balance. Six individual plants were selected for biomass measurement for each of *M. azedarach* and *L. lucidum* species at the beginning of the treatment, respectively, as initial dry weight (DW). The biomass of each component was determined as initial dry weight (DW). After 425 days of treatment (from June 15^th^, 2017 to September 13^th^, 2018), all examined plants were harvested and the biomass was measured as final DW. The determination of relative growth rate (RGR) is based on the method of Environment Canada (2007) guidelines as follows:

$$RGR=(ln\;X_{j}-\ln X_{i})/(t_{j}-t_{i});$$

where *X_i_* and *X_j_* represent the values of final DW and initial DW, respectively; *t_j_* and *t_i_* represent initial time and final time, respectively.

### Gas Exchange Measurements

Three new, similar size and fully expanded leaves per plant were chosen for the measurements of photosynthesis for each treatment. The leaf gas exchange and Chl fluorescence were measured by using an open gas exchange system (LI-COR 6400XT, Lincoln, USA) at the same time with an integrated Chl fluorescence chamber head in the morning (8:00–10:00 am). For all measurements, the following conditions were set up: leaf temperature of 25–32°C, PAR of 1,000 μmol (photon) m^−2^ s^−1^, and vapor pressure deficit (VPD) of 2.0 ± 0.2 kPa.

For developing the relationships between leaf photosynthesis and intercellular CO_2_ concentrations (A–Ci curves), the steady-state rates of leaf maximum net photosynthesis rate (P_n_, μmol m^−2^ s^−1^), stomatal conductance (Gs, mol m^−2^ s^−1^) and intercellular CO_2_ concentration (Ci, μmol m^−2^ s^−1^) were measured. The A–Ci curves were developed by measuring P_n_ at 15 reference CO_2_ concentrations: 400, 300, 250, 200, 150, 100, 50, 25, 0, 400, 600, 800, 1,000, 1,200, 1,400 μmol mol^−1^ as described by Centritto et al. (2003). The maximum carboxylation rate allowed by Rubisco (V_cmax_), day respiration (R_d_), and electron transfer rate of photosynthesis based on NADPH requirement (J_max_) were estimated based on the modeling methods (Farquhar et al., 1980; Sharkey et al., 2007).

Mesophyll conductance to CO_2_ (Gm) was calculated by using ‘variable J method’ (Harley et al., 1992; Sagardoy et al., 2010) as:

(1) $$Gm=P_{n}/(Ci)(\Gamma *(J_{flu}+8(P_{n}+R_{d}))/((J_{flu})4(P_{n}+R_{d}))$$

where, P_n_ and Ci were obtained from gas exchange measurements under saturating light, Γ∗ was the CO_2_ concentration at the compensation point in the absence of mitochondrial respiration and was obtained according to Bernacchi et al. (2002), R_d_ was calculated based on the A–Ci curve on the same leaf according to Pinelli and Loreto (2003).

J_flu_ represented the rate of electron transport and was calculated as:

(2) $$J_{flu}=0.5\cdot \varphi PSII\cdot \alpha \cdot PPFD$$

where, *α* was total leaf absorbance in the visible light range (taken as 0.85, Grassi and Magnani, 2005), 0.5 was a factor to account for the distribution of light between the two photosystems (Laisk and Loreto, 1996). The actual chloroplastic CO_2_ concentration (Cc) was calculated from the g_m_ value as Cc = Ci − (P_n_/Gm) (Harley et al., 1992).

### Photosynthesis Limitation Analysis

The inhibition of Pb–Zn stress on plant photosynthesis was further assessed based on the contribution made by various functional components to the photosynthetic limitations (Sagardoy et al., 2010). The functional components were stomatal (SL), mesophyll conductance (MCL), and leaf biochemical characteristics (BL). The relative contributions of these functional components to the photosynthesis limitations were evaluated when compared with a reference status in which the photosynthesis limitations were ignorable, and the Gs, Gm, and V_cmax_ were at their maximum. In this study, the corresponding values of Gs, Gm, and V_cmax_ taken from C treatments were as reference values; thus the photosynthesis limitations were set to 0.

### The Kinetics of Prompt Fluorescence and Modulated 820 nm Reflection

Fast chlorophyll a fluorescence was measured by using M-PEA (Multifunctional Plant Efficiency Analyser, Hansatech Instrument, UK). After 1 h dark adaptation using dark adaptation clips, leaves were exposed to a pulse of saturating red light (5,000 μmol m^−2^ s^−1^, peak at 625 nm, duration from 50 μs to 2 s, records of 128 points), and the measurements were carried out during a period of 8:30–11:00 am. The OJIP transient was analyzed based on the JIP test (Strasser et al., 2004). The values of fluorescence intensity from the original measurements were used in this study: fluorescence intensity at 20 μs (at the O step, considering as the minimum fluorescence, F_O_); 300 μs (F_300μs_) used for calculation of the initial slope of the relative variable fluorescence kinetics; 2 ms (at the J step, F_J_); 30 ms (at the I step, F_I_), and the P step (considering as the maximum fluorescence, F_m_). The description and calculation of standardization formula of OJIP transients and formula of parameters were listed in **Table 1**.

**Table 1:** Formulae and definitions the technical data of the OJIP curves, the selected JIP-test parameters and the selected PF parameters used in this study.

Technical fluorescence	
F_t_	Fluorescence at time t after onset of actinic illumination
F_O_ ≌ F_20μs_ or F_50μs_	Minimal fluorescence, when all PSII RCs are open
F_K_ = F_300μs_	Fluorescence intensity at the K-step (300 μs) of OJIP
F_J_ = F_20ms_	Fluorescence intensity at the J-step (2 ms) of OJIP
F_I_ = F_30ms_	Fluorescence intensity at the I-step (30 ms) of OJIP
F_P_(=F_M_)	Maximal recorded fluorescence intensity, at the peak P of OJIP
N	the turnover number of QA reduction events
F_υ_ = F_t_ − F_O_	Variable fluorescence at time t
F_V_ = F_M_ − F_O_	Maximal variable fluorescence
V_I_ = (F_M_ − F_I_)/(F_M_ − F_O_)	Relative variable fluorescence at time t
V_K_ = (F_K_ − F_O_)/(F_M_ − F_O_)	Relative variable fluorescence at the K-step
V_J_ = (F_J_ − F_O_)/(F_M_ − F_O_)	Relative variable fluorescence at the J-step
W_K_ = W_300μs_ = (F_300μs_ − F_O_)/(F_J_ − F_O_)	Relative variable fluorescence at the K-step to the amplitude F_J_-F_O_
*φ*_Po_ = PHI(P_0_) = TR_0_/ABS = 1 − F_O_/F_M_	Maximum quantum yield for primary photochemistry
*ψ*_Eo_ = PSI_0_ = ET_0_/TR_0_ = (1 − V_J_)	Probability that an electron moves further than QA^−^
φ_E_o = PHI(E_0_) = ET_0_/ABS = (1 − F_O_/F_M_)/(1 − V_J_)	Quantum yield for electron transport (ET)
*φ*_Ro_ = RE_0_/ABS = *φ*_Po_ × *ψ*_Eo_ × *δ*_Ro_ = φ_Po_ × (1 − V_I_)	Quantum yield for reduction of the end electron acceptors on the PSI acceptor side (RE)
*δ*_Ro_ = RE_0_/ET_0_ = (1 − V_I_)/(1 − V_J_)	Probability that an electron is transported from the reduced intersystem electron acceptors to the final electron acceptors of PSI (RE)
ABS/CS = CH1/CS	Absorption flux per CS
RC/CS = *φ*_P_o × (V_J_/M_0_) × (ABS/CS)	Q_A_-reducing RCs per CS
$PI_{ABS}=\frac{\gamma RC}{1-\gamma RC}\times \frac{\varphi Po}{1-\varphi Po}\times \frac{\varphi Eo}{1-\varphi Eo}$	Performance index (potential) for energy conservation from photons absorbed by PSII to the reduction of intersystem electron acceptors
*Δ*V_IP_ = 1 − V_I_	Amplitude of the I to P phase of the OJIP fluorescence transient (associated with PSI reaction center content).
Abbreviations	
P_n_	net photosynthetic rate
Gs	stomatal conductance
Ci	intercellular CO_2_ concentration
Gm	mesophyll conductance to CO_2_
Rd	day respiration
Cc	chloroplastic CO_2_ concentration
V_cmax_	Maximum carboxylation rate allowed by Rubisco
J_max_	rate of photosynthetic electron transport based on NADPH requirement
SL	stomatal conductance
MCL	mesophyll conductance
BL	biochemical limitation
MDA	malondialdehyde
SOD	superoxide dismutase
POD	peroxidase
CAT	Catalase

Modulated 820 nm reflection was also expressed as MR/MR_O_. The value of MR and MR_O_ were determined by the method of Zhou et al. (2019). The PSI redox was denoted by V_PSI_ (maximum PSI oxidation rate) and V_PSII–PSI_ (maximum PSI reduction rate, respectively, and were obtained according to Gao et al. (2014).

### Evaluation of Chlorophyll and Carotenoid Contents

Leaf chlorophylls and carotenoid content were extracted using 95% ethanol and then placed in the dark for at least 72 h at 4°C. The extracts were measured at wavelengths of 665, 649, and 470 nm spectrophotometrically and was then calculated as milligram per gram of fresh weight (Ji et al., 2018).

### Assay of Superoxide Anion (O_2_^−^) Production Rate, H_2_O_2_ Content, and Malondialdehyde (MDA)

The O_2_^−^ production rate was measured using a reagent kit (Beijing Solarbio Science and Technology, China). H_2_O_2_ content was measured using the method of Okuda et al. (1991). The MDA level was assayed as a thiobarbituric acid reactive substance according to the method of Dhindsa et al. (1981). A 0.25 g fresh leaf sample was homogenized; supernatant was collected and measured at the wave length of 532, 600, and 450 nm using a UV/visible spectrophotometer.

### Estimation of Proline and Antioxidative Enzymes

Proline content was determined using Pure Pro as a standard (Gao, 2006). A 0.1 g fresh leaf sample was homogenized with 5 ml of 3% aqueous sulfosalicylic acid and was then extracted in a boiling bath.

For determination of SOD, POD, and CAT, a 0.3 g fresh leaf sample was grounded in 3 ml extraction buffer containing 25 mM Hepes, 2% polyvinyl-pyrrolidone (PVP), 0.2 mM EDTA, and 2 mM ascorbate (pH 7.8) and was centrifuged at 12,000 g for 20 min at 4°C. The supernatants were collected for enzyme analysis.

SOD activity was determined according to Giannopolitis and Ries (1977). The 3 ml reaction mixture contained 0.05 M phosphate buffer solution (pH 7.8), 0.06 M Riboflavin, 0.195 M Met, 0.003 M EDTA, 1.125 mM NBT, and 0.2 ml enzyme supernatants. The measurements were performed at 25°C. The tested samples were incubated for 10–20 min under 10,000 lx irradiance; inhibition rate of nitro blue tetrazolium (NBT) reached to 50% represented one unit of SOD activity as by spectrophotometer at 560 nm.

POD activity was assessed as in Beffa et al. (1990). The reaction mixture contained 0.1 M sodium-acetic buffer (pH 5.0), 0.25% (w/v) guaiacol, 0.75% H_2_O_2_, and 0.05 ml enzyme supernatants. One unit of POD activity was represented as an increase of 0.01 *Δ*OD value a minute at 470 nm.

CAT activity was determined according to Aebi (1984). The reaction mixture contained 0.05 M phosphate buffer (pH 7.0) and 0.45 M H_2_O_2_ and 0.2 ml enzyme supernatants. One unit of CAT activity was represented as decrease of *Δ*OD value a minute at 240 nm. The measurement for each antioxidative enzyme was repeated three times.

### Assay of Leaf Total Rubisco Activity

Ribulose-1, 5-bisphosphate carboxylase/oxygenase (Rubisco) of leaves from the tested plants was assayed according to the method of Chen et al. (2005). After centrifugation at 13,000 r·min^−1^ 40 s at 2°C, the supernatant was used immediately for assays of Rubisco activity (Jiang et al., 2008).

### Total Metal Content

For the determination of the metal contents in plant, the different dried plant components were powdered and passed through an 80-mesh sieve. About 0.1 g of plant material was digested with 1 ml of HNO_3_ and H_2_O_2_ (8:2, v/v). Then, the tube with plant material was on the block at 200°C for 0.75–1 h. The residue was taken up in 10 ml of demineralized water. The concentrations of Pb and Zn were determined by atomic absorption spectrophotometry (AA-6800, Shimadzu, Kyoto, Japan) (Huang et al., 2019). The measurement for each sample was repeated six times, and the mean value was calculated.

The metal translocation factor (TF) in the leaves was represented as the metal concentration ratio of plant leaves and stems to roots (Han et al., 2016).

### Statistical Analyses

Two-way analysis of variance (ANOVA) was performed on the data using SPSS (20.0). Differences among the eight treatment combinations (two species × four Pb–Zn mine tailing treatments) were analyzed by two-way analysis of variance; eight means were separated by Duncan’s new multiple range test at P < 0.05 level. Data were presented as means ± SD (n = 6). The principal component analysis (PCA) used CANOCO version 5.0.

## Results

### RGR, Pb/Zn Contents in Roots, Stems, and Leaves

As Pb–Zn tailing portions increased, RGR of *M. azedarach* and *L. lucidum* was decreased progressively compared with the control group (*P* < 0.05) (**Table 2**), the RGR values were reduced by 10–90% and 6–70% in *M. azedarach* and *L. lucidum*, respectively, in Pb–Zn treatments when compared with the C plants (**Table 2**). The concentrations of Pb and Zn in the leaves, stems, and roots increased with the increase of the proportion of Pb–Zn tailings in both tested plants compared to the C (**Table 2**). The Pb and Zn concentrations were significantly higher in the roots than in the leaves and stems. The Pb and Zn concentrations showed as Zn > Pb in the leaves, stems, and roots in all Pb–Zn tailing treatments. TFs of Pb and Zn were higher in *M. azedarach* than those in *L. lucidum*.

**Table 2:** Relative growth rate (RGR), Pb and Zn concentrations and translocation coefficient of *M. azedarach* and *L. lucidum* grown in soil mixed with different proportions of Pb–Zn mine tailings.

			RGR	Pb content				Zn content			
				Root	Leaf	Stem	TF	Root	Leaf	Stem	TF
*M. azedar-ach*	C		0.010 ± 0.002a	0.57 ± 0.04a	0.31 ± 0.06a	0.15 ± 0.05a	0.80 ± 0.27a	27.32 ± 1.98a	30.36 ± 1.56a	31.44 ± 0.91a	2.26 ± 0.22a
	L1		0.009 ± 0.004a	15.93 ± 0.41b	9.68 ± 0.78b	8.01 ± 0.35b	1.11 ± 0.31b	49.12 ± 2.32b	70.5 ± 3.34b	33.88 ± 1.05a	2.13 ± 0.35a
	L2		0.005 ± 0.003b	43.96 ± 2.18c	26.08 ± 2.56c	13.67 ± 3.32c	0.90 ± 0.16a	101.49 ± 3.22c	126.41 ± 4.78c	41.81 ± 2.25b	1.66 ± 0.27b
	L3		0.001 ± 0.000c	105.13 ± 6.83d	50.98 ± 1.45d	16.39 ± 0.41c	0.65 ± 0.22a	236.14 ± 7.63d	177.88 ± 8.34d	62.15 ± 3.04c	1.01 ± 0.18c
*L. lucidu-m*	C		0.018 ± 0.002a	0.31 ± 0.04a	0.41 ± 0.04a	0.22 ± 0.02a	2.02 ± 0.11a	24.15 ± 2.02a	28.46 ± 0.57a	24.36 ± 1.12a	2.19 ± 0.33a
L1		0.017 ± 0.001b	20.84 ± 1.22b	5.48 ± 0.63b	5.41 ± 0.22b	0.52 ± 0.09b	69.51 ± 4.41b	50.32 ± 4.32b	33.12 ± 0.76b	1.20 ± 0.25b	
L2		0.009 ± 0.002c	59.78 ± 3.54c	15.64 ± 1.15c	8.46 ± 0.76c	0.40 ± 0.12b	158.45 ± 5.67c	78.16 ± 4.09c	36.39 ± 1.77b	0.72 ± 0.21b	
L3		0.005 ± 0.003d	141.04 ± 4.32d	36.75 ± 1.93d	9.13 ± 0.55c	0.33 ± 0.04b	288.12 ± 8.91d	99.65 ± 3.88d	51.12 ± 2.09c	0.53 ± 0.11c	

TF = the metal concentration ratio of plant leaves and stems to roots. Data are means ± SE, n = 6. Values with different letters within the same column indicate significant differences at the p < 0.05 level between Pb–Zn treatments. C: 100% garden soil, L1: 90% garden soil + 10% Pb–Zn tailings, L2: 75% garden soil + 25% Pb–Zn tailings, L3: 50% garden soil + 50% Pb–Zn tailings.

### Pigment Contents

**Figure 1:**
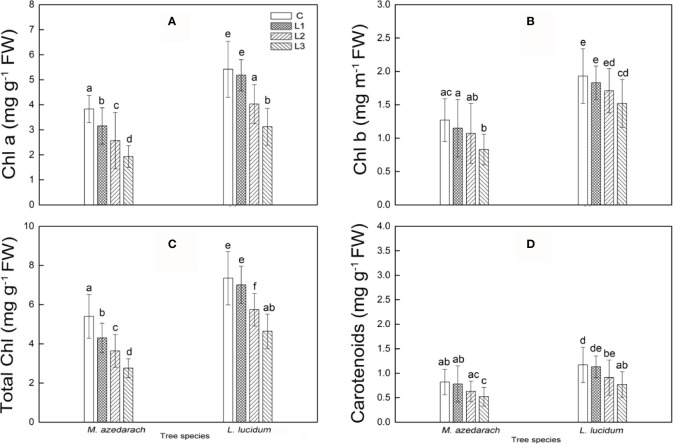
Chlorophyll a **(A)**, chlorophyll b **(B)**, total chlorophyll **(C)** and carotenoids **(D)** in leaves of *M. azedarach* and *L. lucidum* grown in soil mixed with different proportions of Pb–Zn mine tailings. C (control), L1, L2, and L3 represent 100% garden soil, 90% garden soil + 10% Pb–Zn tailings, 75% garden soil + 25% Pb–Zn tailings, 50% garden soil + 50% Pb–Zn tailings, respectively. Different letters above the bars indicate a significant difference at P < 0.05. Values are means of n = 6; bar indicates standard error.

The photosynthetic pigment contents of the two tested plants were found to decline with increasing Pb–Zn tailing portions (**Figures 1A–D**). A significant reduction in chlorophylls a, b, and total chlorophyll in the two tested plants was observed under Pb–Zn tailing stress as compared to the C, respectively. Carotenoid content was notably affected by Pb–Zn tailing stress when the Pb–Zn treatment exceeded a portion of 10%. Compared with *L. lucidum*, *M. azedarach* showed a faster increase and a greater extent of chlorophylls a, b, total chlorophyll, and carotenoid content. When compared to the C groups, Chl a decreased by a range of 18 to 50%, Chl b from 9 to 35%, total chlorophyll from 16 to 46%, and the carotenoids from 5 to 37% in *L. lucidum* in L1–L3 treatments. The corresponding values decreased in *M. azedarach* from 4to 42% in Chl a, from 5 to 21% in Chl b, from 5 to 37% in total chlorophyll, and from 3 to 34% in carotenoids.

### Gas Exchange

**Figure 2:**
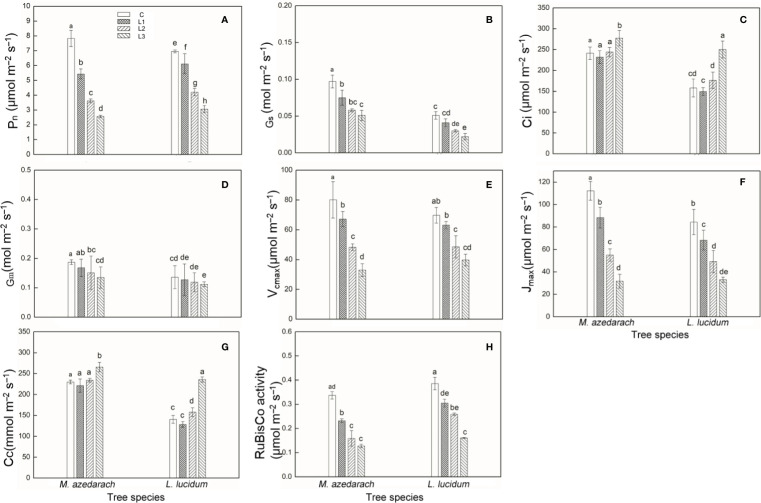
Photosynthetic parameters measured with a Li-6400 gas exchange system and rubisco activity in *M. azedarach* and *L. lucidum* grown in soil mixed with different proportions of Pb–Zn mine tailings. **(A)** Pn, net photosynthetic rate; **(B)** Gs, stomatal conductance; **(C)** Ci, intercellular CO_2_ concentration; **(D)** Gm, mesophyll conductance to CO_2_; **(E)** V_cmax_, maximum carboxylation rate allowed by Rubisco; **(F)** J_max_, rate of photosynthetic electron transport based on NADPH requirement; **(G)** Cc, chloroplastic CO2 concentration; **(H)** Rubisco activity. C (control), L1, L2, and L3 represent 100% garden soil, 90% garden soil + 10% Pb–Zn tailings, 75% garden soil + 25% Pb–Zn tailings, 50% garden soil + 50% Pb–Zn tailings, respectively. Different letters above the bars indicate a significant difference at P < 0.05. Values are means of *n* = 6; bar indicates standard error.

The Pb–Zn tailing treatments had an inhibitory effect on Pn, Gs, Gm, V_cmax_, J_max_, and Rubisco activity, but had promotion effect on Ci and Cc in both examined plants (**Figure 2**). Pn, V_cmax_, J_max_ significantly decreased after Pb–Zn mine tailing treatment. The Gm of the leaves from the two plants gradually decreased with the increase of Pb–Zn tailing portions. For *M. azedarach*, Gs showed a significant difference between C and L1 treatments and then gradually decreased when the plants were grown in L2 treatments, and eventually reached the minimum value at L3 treatments. In *L. lucidum*, Gs was decreased slightly as Pb–Zn tailing portions increased. Both plants exhibited the similar tendency in Ci and Cc as Pb–Zn tailing portions increased. Ci and Cc decreased slightly when the plants were in L1 treatments and then significantly increased in L2 and L3 treatments (*P* < 0.05), but the change of Ci was higher in *L. lucidum* (increased from −5 to 58%) than in *M. azedarach* (increased from −4 to 5%). The decrease of P_n_, Gm, V_cmax_, and J_max_ in *M. azedarach* showed a greater extent than that in *L. lucidum*. When compared to the C groups, the Pn decreased by a range of 12 to 56%, the Gm from 7 to 18%, the V_cmax_ from 9 to 43%, the J_max_ from 19 to 64%, and the Rubisco activity from 10 to 52% in *L. lucidum* in L1–L3 treatments. The corresponding values decreased in *M. azedarach* from 31 to 67% in Pn, from 10 to 28% in Gm, from 16 to 59% in V_cmax_, from 21 to 72% in J_max_, and from 31 to 62% in the Rubisco activity.

Under Pb–Zn tailing treatments, biochemical limitations (BLs) increased in the two tested plants. The BL values of *M. azedarach* increased 12, 34, and 53% in the L1, L2, and L3 treatments compared to the C, respectively (**Table 3**). The corresponding values increased 6, 23, and 36% in L1, L2, and L3 treatments in *L. lucidum*, respectively. The stomatal conductance limitation (SL) and the mesophyll conductance (MCL) of leaves in both trees increased slightly under Pb–Zn treatments. The SL values increased 4–6% and the MCL values increased 0.4–0.8% for *M. azedarach* and *L. lucidum* when compared to C.

**Table 3:** Photosynthesis limitation parameters (%) in *M. azedarach* and *L. lucidum* grown in soil mixed with different proportions of Pb–Zn mine tailings.

	C	Pb–Zn tailings treatments		
		L1	L2	L3
*M. azedarach*				
SL	0.0%	5.1%	5.6%	4.0%
MCL	0.0%	0.5%	0.5%	0.8%
BL	0.0%	12.2%	33.6%	53.1%
*L. lucidum*				
SL	0.0%	5.0%	6.0%	6.4%
MCL	0.0%	0.4%	0.5%	0.4%
BL	0.0%	5.8%	23.1%	35.9%

Data are means ± SE, n = 6. The control (C) was taken as a reference, for which all limitations were set to 0. SL: stomatal conductance limitation, MCL: mesophyll conductance, BL: biochemical limitations. L1: 90% garden soil + 10% Pb–Zn tailings, L2: 75% garden soil + 25% Pb–Zn tailings, L3: 50% garden soil + 50% Pb–Zn tailings.

### Prompt Fluorescence OJIP Transient Analysis

**Figure 3:**
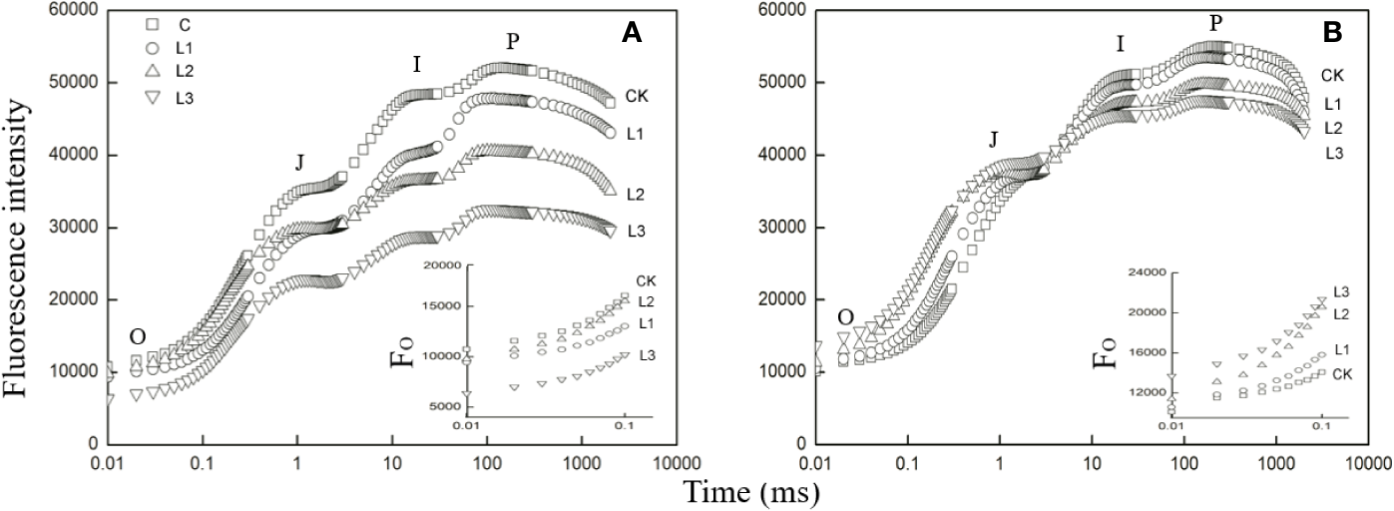
Fast induction curves of chlorophyll fluorescence (OJIP) in *M. azedarach* **(A)** and *L. lucidum* **(B)** grown in soil mixed with different proportions of Pb–Zn mine tailings. The letters O, J, I, and P refer to the selected time points used by the JIP-test for the calculation of structural and functional parameters. The used signals are: the fluorescence intensity at F_O_≌F_20μs_ or F_50μs_; at 3 ms = F_J_; and at 30 ms = F_I_; the maximal fluorescence intensity, F_P_ = F_M_. C (control); L1, L2, and L3 represent 100% garden soil, 90% garden soil + 10% Pb–Zn tailings, 75% garden soil + 25% Pb–Zn tailings, 50% garden soil + 50% Pb–Zn tailings, respectively. In the insets of the two panels, the F_O_ of the fluorescence rise is compared between measurements on control and Pb–Zn treated leaves.

**Figure 4:**
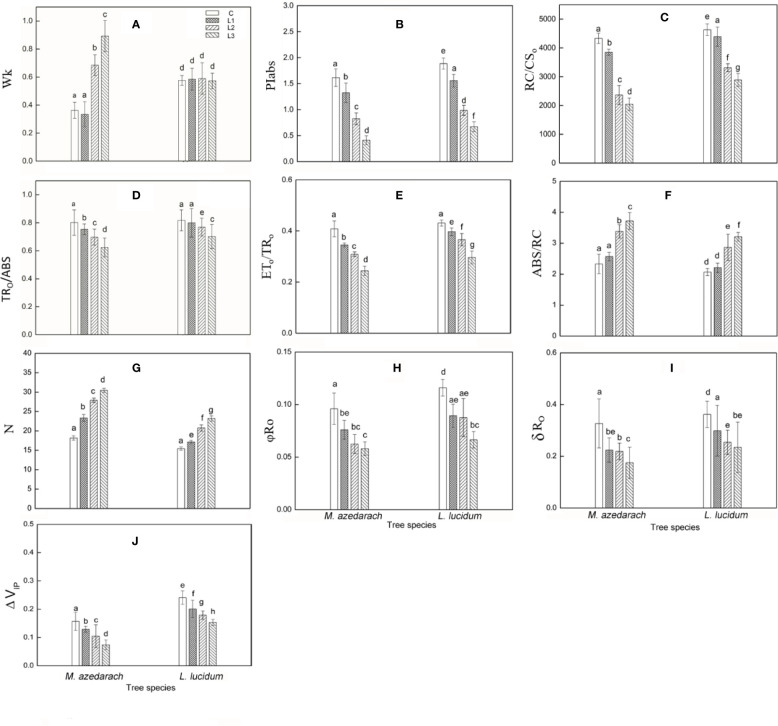
Parameters derived from OJIP transients of *M. azedarach* and *L. lucidum* grown in soil mixed with different proportions of Pb–Zn mine tailings. **(A)** W_K_,relative variable fluorescence at the K-step to the amplitude F_J_–F_O_; **(B)** PI_ABS_, performance index (potential) for energy conservation from photons absorbed by PSII to the reduction of intersystem electron acceptors; **(C)** RC/CS_O_, the density of PSII reaction center (RC) per excited cross-section (at t = 0); **(D)** TR_O_/ABS, maximum quantum yield for primary photochemistry; **(E)** ET_O_/TR_O_, probability that an electron moves further than QA^−^; **(F)** ABS/CS, absorption flux per unit area; **(G)** N, the turnover number of Q_A_ reduction events; **(H)** *φ*_Ro_, quantum yield for reduction of the end electron acceptors on the PSI acceptor side (RE); **(I)** *δ*_Ro_, probability that an electron is transported from the reduced intersystem electron acceptors to the final electron acceptors of PSI (RE); **(J)** *Δ*V_IP_, amplitude of the I to P phase of the OJIP fluorescence transient (associated with PSI reaction center content). C (control); L1, L2, and L3 represent 100% garden soil, 90% garden soil + 10% Pb–Zn tailings, 75% garden soil + 25% Pb–Zn tailings, 50% garden soil + 50% Pb–Zn tailings, respectively. Different letters above the bars indicate a significant difference at P < 0.05. Values are means of *n* = 6, bar indicates standard error.

The Pb–Zn stress had a considerable effect on fluorescent OJIP transients in two tested plants (**Figure 3**). The F_o_ values were significantly reduced under Pb–Zn treatments in *M. azedarach* compared to the C (**Figure 3A**, inset). However, the F_o_ values of *L. lucidum* had no significant increase under Pb–Zn tailing treatments compared to the C (**Figure 3B**, inset). Three Pb–Zn tailing treatments did not affect W_k_ in *L. lucidum* compared to the C (**Figure 4A**). In *M. azedarach*, W_k_ was similar for both C and L1 treatments and significantly increased in L2 and L3 treatments (**Figure 4A**). Pb–Zn stress treatments significantly decreased performance index (PIabs) in *M. azedarach* and *L. lucidum* (**Figure 4B**) but distinctly increased the turnover number of Q_A_ reduction events (N) and absorption flux (ABS/RC) (**Figures 4F, G**). Meanwhile, RC/CS_O_, TR_O_/ABS, and ET_O_/TR_O_ were decreased dramatically by Pb–Zn stress (**Figures 4C–E**).

The value of *φ*R_o_, *δ*R_o_ and *Δ*V_IP_ significantly decreased in *M. azedarach* and *L. lucidum* as Pb–Zn tailing portions increased (**Figures 4H–J**). When compared to the C groups, the *φ*R_o_ decreased by 21, 34, and 40%, the *δ*R_o_ decreased by 32, 33, and 47%, and the *Δ*V_IP_ decreased by 18, 33, and 53% in *M. azedarach* in L1, L2, and L3 treatments, respectively. The corresponding values decreased 19, 24, and 36% in *φ*R_o_, 17, 30 and 35 in *δ*R_o_, 17, 26, and 37% in *Δ*V_IP_ in *L. lucidum*.

### MR/MR_O_ Transient Analysis

**Figure 5:**
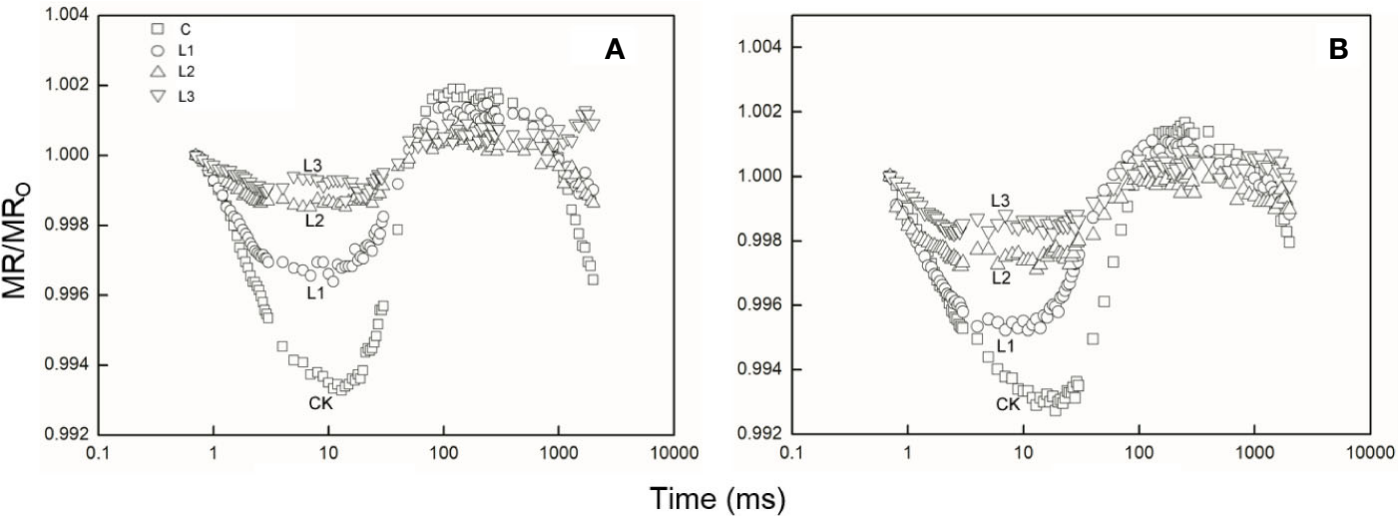
The 820 nm light reflection curves of *M. azedarach* **(A)** and *L. lucidum* **(B)** in soil mixed with different proportions of Pb–Zn mine tailings. Modulated 820 nm reflection was expressed as MR/MR_O_, where MR_O_ is the modulated reflection value at the onset of actinic illumination (taken at 0.7 ms, the first reliable MR measurement) and MR is the modulated reflection signal during illumination. C (control); **(A)** L1, **(B)** L2, and **(C)** L3 represent 100% garden soil, 90% garden soil + 10% Pb–Zn tailings, 75% garden soil + 25% Pb–Zn tailings, 50% garden soil + 50% Pb–Zn tailings, respectively.

After Pb–Zn tailing treatments, the shape of the MR/MR_O_ kinetics was obviously changed in the two tested plants. The lowest points represented a turning point of PSI oxidation state (**Figure 5**). When compared to the C, the lowest points on the reflection curve of both tested plants increased with rising Pb–Zn tailing portions. The V_PSI_ and V_PSII–PSI_ of both tested plants declined significantly with the rising Pb–Zn tailing portions (**Table 4**). V_PSI_ and V_PSII–PSI_ of both tested plants significantly decreased in the treated groups than in the C groups. Compared with *L. lucidum*, *M. azedarach* showed a greater decrease of V_PSI_ and V_PSII–PSI_.

**Table 4:** Parameters derived from the modulated 820-nm reflection (MR/MR_O_) of the *M. azedarach* and *L. lucidum* grown in soil mixed with different proportions of Pb–Zn mining tailings.

M. azedarach	V_PSI_	V_PSII-PSI_	V_PSII_
C	2.065 ± 0.212a	0.095 ± 0.008a	2.160 ± 0.145a
L1	1.309 ± 0.088b	0.063 ± 0.014b	1.372 ± 0.056b
L2	0.561 ± 0.107c	0.019 ± 0.007c	0.580 ± 0.009c
L3	0.413 ± 0.069d	0.005 ± 0.000d	0.418 ± 0.011d
*L. lucidum*			
C	2.046 ± 0.054a	0.036 ± 0.004a	2.082 ± 0.094a
L1	1.544 ± 0.131b	0.021 ± 0.006b	1.565 ± 0.083b
L2	1.804 ± 0.009c	0.010 ± 0.002c	1.814 ± 0.115c
L3	0.681 ± 0.102d	0.008 ± 0.001d	0.699 ± 0.057d

Data are means ± SE, n = 6. Different small letters in the same column indicate significant difference at 0.05 level by Duncan’s new multiple test. V_PSI_: maximum slope decrease of MR/MR_O_; V_PSII–PSI_: maximum slope increase of MR/MR_O_; V_PSII_ = V_PSI_ + V_PSII–PSI_. L1 90% garden soil + 10% Pb–Zn tailings, L2: 75% garden soil + 25% Pb–Zn tailings, L3: 50% garden soil + 50% Pb–Zn tailings.

### PI_abs_, *Δ*V_IP_ in Relation to V_cmax_

**Figure 6:**
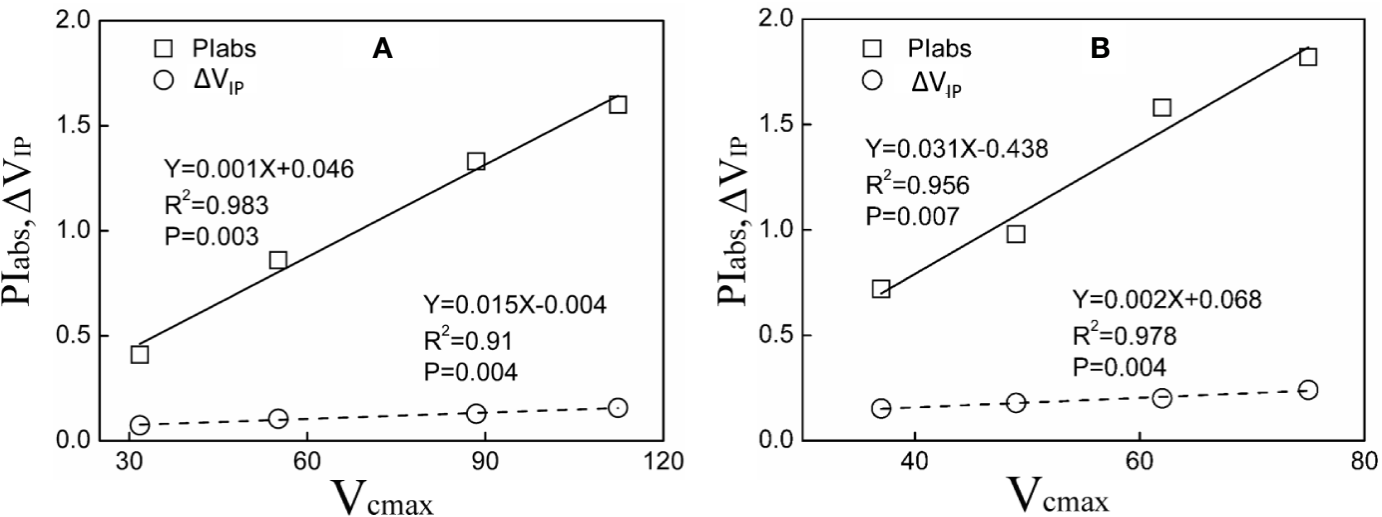
Relationship between PIabs, *Δ*V_IP_ and V_cmax_ for *M. azedarach* **(A)** and *L. lucidum* **(B)** in soil mixed with different proportions of Pb–Zn mine tailings.

A significant positive relationship of PI_abs_, *Δ*V_IP_ and V_cmax_ was observed in both tested plants under Pb–Zn mine tailing treatments (**Figure 6**). As V_cmax_ decreased, PI_abs_, *Δ*V_IP_ decreased linearly in *M. azedarach* and *L. lucidum*, respectively.

### O_2_^−^ Production Rate, H_2_O_2_, MDA and Proline Content in Leaves

**Figure 7:**
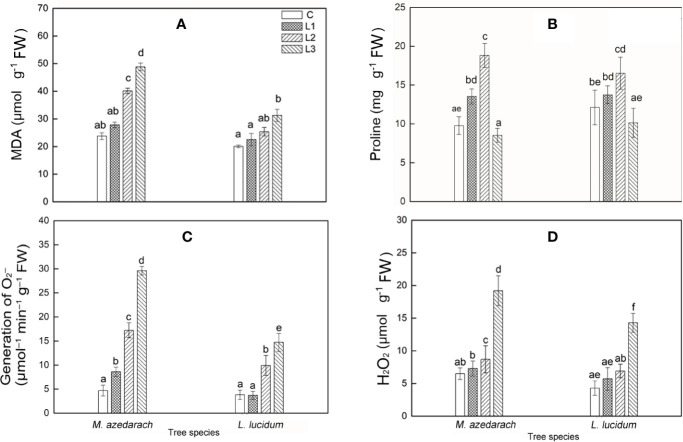
Malondialdehyde (MDA) **(A)**, Proline **(B)**, O_2_**^−^** production rate **(C)**, H_2_O_2_ content **(D)** in leaves of *M. azedarach* and *L. lucidum* grown in soil mixed with different proportion of Pb–Zn mine tailings. C (control); L1, L2, and L3 represent 100% garden soil, 90% garden soil + 10% Pb–Zn tailings, 75% garden soil + 25% Pb–Zn tailings, 50% garden soil + 50% Pb–Zn tailings, respectively. Different letters above the bars indicate a significant difference at P < 0.05. Values are means of *n* = 6; bar indicates standard error.

With an increase of Pb–Zn mine tailing portions, the O_2_^−^ production rate, H_2_O_2_, and MDA contents of two tested plant leaves increased notably (**Figures 7A, C, D**). Compared with *L. lucidum*, *M. azedarach* showed a faster increase and a greater extent O_2_^−^ production rate, H_2_O_2_, and MDA contents. The proline content in *M. azedarach* and *L. lucidum* reached the peak at L2 treatments (**Figure 7B**), where the proline content was 1.96 and 1.36 times higher than that in the C treatments, respectively. Under L3 treatments, the proline content in *M. azedarach* and *L. lucidum* decreased by 16 and 13% of that in the C, respectively.

### Antioxidant Enzyme Activity in Leaves

**Figure 8:**
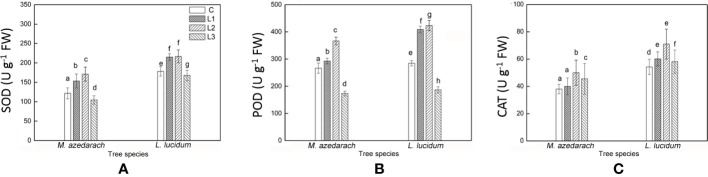
Superoxide dismutase (SOD) **(A)**, Peroxidase (POD) **(B)** and Catalase (CAT) **(C)** in leaves of *M. azedarach* and *L. lucidum* grown in soil mixed with different proportions of Pb–Zn mine tailings. C (control); L1, L2, and L3 represent 100% garden soil, 90% garden soil + 10% Pb–Zn tailings, 75% garden soil + 25% Pb–Zn tailings, 50% garden soil + 50% Pb–Zn tailings, respectively. Different letters above the bars indicate a significant difference at P < 0.05. Values are means of *n* = 6; bar indicates standard error.

The SOD, POD, and CAT displayed similar trends in activity in both tested trees with the increase of Pb–Zn mine tailing portions (**Figures 8A–C**). Compared with the C, the activities of SOD and POD in both tested trees significantly increased under L1 and L2 treatments and decreased under L3 treatment. The CAT activity in both tested trees under all Pb–Zn treatments was significantly higher than that in the C plants. Notably, the activities of SOD, POD, and CAT in *L. lucidum* were significantly higher than that in *M. azedarach* under all Pb–Zn mine tailing treatments.

### Principal Component Analysis

**Figure 9:**
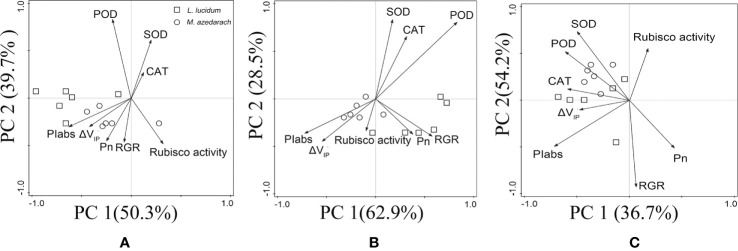
Principal component analysis (PCA) of antioxidant enzymes, PSII performance, PSI content, net photosynthesis rates (Pn), and the relative growth rate (RGR) of *M. azedarach* and *L. lucidum* exposed to different proportions of Pb–Zn mine tailings. **(A)** L1, **(B)** L2, and **(C)** L3 represent 100% garden soil, 90% garden soil + 10% Pb–Zn tailings, 75% garden soil + 25% Pb–Zn tailings, 50% garden soil + 50% Pb–Zn tailings, respectively.

PCA was used to understand Pb–Zn tailings affecting the photosynthetic characteristics including RGR, PSII performance, PSI content, net photosynthesis rates (Pn), and the antioxidative enzymes of leaves in both trees (**Figure 9**). The first two components comprised 90.0% (50.3% for PC1 and 39.7% for PC2), 91.4% (62.9% for PC1 and 28.5% for PC2), and 90.9% (36.7% for PC1 and 54.2% for PC2) of the total variations in L1, L2, and L3 treatments, respectively. Under L1 and L2 treatments, PIabs, *Δ*V_IP_, and Pn were the most influential in the PC1; and SOD, POD and CAT in the PC2. Under L3 treatments, SOD, POD, and CAT were the most influential in the PC1, and PIabs and *Δ*V_IP_ in the PC2. Evidently, two tested plans were more closely to photosynthetic parameters (PIabs, ΔV_IP_, and Pn) than to antioxidative enzymes (SOD, POD and CAT) under L1 and L2 treatments, but were more closely to antioxidative enzymes than to photosynthetic parameters under L3 treatment. In addition, RGR was positively related to the photosynthetic parameters in the tested plants.

## Discussion

### The Reduction of Plant Growth Is Attributed to the Depression of Photosynthesis

When plants were grown in Pb–Zn contaminated soils, plant growth and development were retarded and eventually the biomass production decreased (Ha et al., 2011; Han et al., 2013). In this study, the RGR values for the two tested trees decreased under Pb–Zn treatments (**Table 2**). It was well known that plant biomass productivity was dependent on the photosynthetic assimilating accumulation which provided energy and carbon sources (Liu et al., 2017). A positive correlation was found between Pn and RGR in this study (**Figure 9**), suggesting the photosynthetic processes were inhibited by Pb–Zn stress in the tested tree species.

### The Photosynthesis Limitation Is Mainly Attributed to the Biochemical Limitation

A significant decrease of gas exchange parameters was observed in the Pb–Zn treated plants (**Figure 2**). Photosynthesis limitations might be derived from different physiological processes, such as stomatal opening, mesophyll conductance to CO_2_, and the carboxylation capacity (Flexas et al., 2008; Centritto et al., 2009). In this study, although Gs and Gm significantly decreased (**Figures 2B, D**), they probably played a minor role in limiting photosynthesis because Ci and Cc increased simultaneously (**Table 3**, **Figures 2C, G**). The biochemical limitation was likely the major factor affecting photosynthesis in *M. azedarach* and *L. lucidum* (**Table 3**). Change for the results were in line with the findings of other studies (Seregin and Kozhevnikova, 2006; Velikova et al., 2011). The biochemical limitations were related to the considerable declines in V_cmax_ and J_max_ with increasing Pb–Zn stresses. The *M. azedarach* suffered more metabolic damage than that in *L. lucidum* (**Figures 2E, F**), indicating that the supply of energy source and carbon skeleton for plant growth and development was lower in *M. azedarach* than in *L. lucidum* (Imsande and Touraine, 1994). The decrease of V_cmax_ values might be ascribed to a reduction of Rubisco activity in the photosynthetic process (**Figure 2H**). The influence of Rubisco activity due to adverse environmental conditions has been reported by other studies (Bah et al., 2010). The J_max_ inhibition in the leaves under Pb–Zn tailing treatments observed from the changes in A/Cc curves indicated the photochemical limitation occurred in our study. The results were in line with the finding which Velikova et al. (2011) reported that alterations in the electron transport rate from PSII to PSI under heavy metals stresses result in a J_max_ limitation.

In addition, there was a decrease in chlorophyll and carotenoid content with the rising Pb–Zn stress in *M. azedarach* and *L. lucidum*, which illustrated that Pb–Zn stress had harmed the photosynthetic apparatus for these two tree species. The decrease of chlorophyll content might be attributed to HMs interfering Fe metabolism, inhibiting chlorophyll synthetase activity and enhancing chlorophyll enzyme activity (Rana, 2015). As a result, chlorophyll degradation ultimately inactivated photosynthesis (Hajihashemi and Ehsanpour, 2013). We found that the decrease degree of chlorophyll content was higher than that of carotenoid content by HMs. The results were in line with the previous finding by Amir et al. (2020) who reported that chlorophylls were more susceptible to the negative impact of HMs as compared to carotenoids.

### The Photosynthetic Electron Transport Is Altered by the Pb–Zn Tailing Stress

Changes in environmental factors could affect photosynthetic performance (Ji et al., 2018). The impacts of HM stresses on the electron transfer and energy balance in photosynthetic processes are quantified by OJIP fluorescence (Li and Zhang, 2015; Guo et al., 2018). In this study, the chlorophyll ﬂuorescence transient curves of the two tested plants were all modified by Pb–Zn mine tailings (**Figure 3**). With the increase of proportions of Pb–Zn mine tailings, F_O_ values were significantly reduced in *M. azedarach*, but increased in *L. lucidum* compared to the control (**Figure 3**); the value of F_P_ decreased in the two tested plants. The F_O_ decrease in *M. azedarach* was likely attributed to the photoinhibitory damage on PSII acceptor (Setlik et al., 1990), and the increase of F_O_ in *L. lucidum* might be caused by increasing amount of free chlorophylls to the PSII RC (Gilmore et al., 1996). The decrease in F_p_ was observed in other heat and salt stress studies due to the increased fraction of inactive RCs (Jedmowski and Brüggemann, 2015; Oukarroum et al., 2015). W_K_ was the donor site parameter of PSII, which has been widely used to analyze damage to OEC by HM stresses (Li and Zhang, 2015). In this study, an increase of W_k_ value in *M. azedarach* (**Figure 4A**) suggested that Pb–Zn stress not only impaired the OEC; the process of electron transfer was also impaired at the P680 donor site (Jiang et al., 2008; Dąbrowski et al., 2016). However, the OEC and the donor side of PSII were not impaired by Pb–Zn stress in *L. lucidum* since W_k_ values did not show a significant change.

Here, several important JIP-test parameters based on the OJIP transient were used to detect and quantify the changes of the photosystem status under Pb-Zn mine tailings stresses. The reduction of the maximum quantum yield of primary photochemistry (TRo/ABS) (**Figure 4D**) and the electron numbers moved further than the Q_A_^−^ (ET_O_/TR_O_) (**Figure 4E**) indicating that Pb–Zn stress resulted in a fast accumulation of Q_A_^−^ in the PSII reaction centers. The results suggested the photosynthetic electron transport was impaired in the acceptor side of PSII, and the electron flow beyond Q_A_^−^ was blocked (Bernardini et al., 2016). It was reported that photoinhibition process can be more accurately assessed by the changes between flux ratios and specific fluxes per reaction center (RC) (Baker and Rosenqvist, 2004; Mlinarić et al., 2017). It was worth noting that the RC/CS_O_ was diminished and ABS/RC was risen in the two tested plants (**Figures 4C, F**), suggesting that although the numbers of active PSII RCs were decreased under Pb–Zn stress, the photochemical reaction efficiency of the remaining RCs was improved by maintaining the regular photosynthesis ability (Paunov et al., 2018). Due to the inactivation of a number of reaction centers, the remaining RCs had to increase their turnover in order to completely reduce the PQ pool (Zhou et al., 2019) as shown by a rise in parameter N (**Figure 4G**). *M. azedarach* had lower values of PIabs, RC/CS_O_, TR_O_/ABS, and ET_O_/TR_O_ and higher values of ABS/RC than that in *L. lucidum*, indicating that Pb–Zn stress may affect, to a greater extent, the PSII activity in *M. azedarach* species.

*Δ*V_IP_ has been used to describe the changes in content of PSI reaction center under detrimental conditions (Oukarroum et al., 2009; Ceppi et al., 2012). The drought and Zn stress conditions declined *Δ*V_IP_ in *Hordeum spontaneum* (Oukarroum et al., 2009) and *Phragmites australis* (Bernardini et al., 2016). In this study, *Δ*V_IP_ was significantly lower for the two plants grown in Pb–Zn contaminated soil when compared to C (**Figure 4J**), indicating that Pb–Zn stress decreased the PSI reaction center in the two tested tree species. The significant reductions of *φ*R_o_ and *δ*R_o_ (**Figures 4H, I**) indicated that the effect of Pb–Zn stress on electron flow occurred in the acceptor side of PSI because of the reduction of the PSI content (Hu et al., 2018). In addition, the values of V_PSI_ of the two tested plants decreased in the Pb–Zn treated groups when compared to the C (**Figure 5**, **Table 4**), indicating that the oxidization reactions were inhibited due to the Pb–Zn stress. The decline in the V_PSII–PSI_ suggested that the reduction activity of PSI activity was injured by the Pb–Zn stress. Therefore, the decreases in maximum oxidization and reduction activity of PSI reaction center were observed since Pb–Zn stress affected PSI content.

In this study, a positive linear relation was found between PI_abs_ and V_cmax_ in this study suggesting that PSII performance was positively related to CO_2_ assimilation (**Figure 6**). The result was in accordance with the suggestions provided by Ghotbi-Ravandi et al. (2014); they reported that the reduction in PSII performance played an important role in the decrease of CO_2_ assimilation rate in *Morocco* under mild and severe drought stresses. Meanwhile, *Δ*V_IP_ also decreased linearly with the decrease of V_cmax_; the result suggested that PSI content might limit the capacity of CO_2_ assimilation (**Figure 6**). Because the decrease of PSI content could disturb the electron flow from PSII to PSI and limited the synthesis of ATP and NADPH (Brestic et al., 2015), the inhibition of CO_2_ assimilation rate can be suggested as a result of the excessive excitation energy that damage the photosystems and especially impeded the photochemical activity of PSI (Zivcak et al., 2013). Specifically, *M. azedarach* suffered more serious eﬀects on the photosynthetic electron transport chain than *L. lucidum* under Pb–Zn stress. This finding was supported by the fact that photosynthetic parameters (PIabs, ΔV_IP_, and Pn) were more affected in *M. azedarach* than that in *L. lucidum* in PCA analysis (**Figure 9**).

### *L. lucidum* May Be More Tolerant to Pb–Zn Stress Than *M. azedarach*

It is previously demonstrated that adverse conditions impaired PSII electron transport; the excess electron resulted in increasing the levels of ROS and consequently caused oxidative stress (Pospíšil, 2009; Guo et al., 2018). The malondialdehyde (MDA) content represents level of lipid peroxidation, which was able to indicate oxidative damage of membrane lipids under stress conditions (Sharma and Dubey, 2005). In this study, with the increase of proportion of Pb–Zn mine tailings, the O_2_**^−^** production rate and H_2_O_2_ content in both tested tree leaves increased significantly, contributing to a significant increase of MDA content. This result was in line with the previous reports where MDA content increased in *Peganum harmala*, *Kandelia obovate*, and *Alternanthera bettzickiana* under HMs stress (Lu et al., 2010; Cheng et al., 2017). In this study, proline contents increased in *M. azedarach* and *L. lucidum* leaves in L1 and L2 treatments but decreased at L3 treatments when compared to the C (**Figure 7B**). The phenomena suggested the increase of proline contents at low and medium Pb–Zn treatments could maintain the integrity of cellular membranes, protect proton pump, and eliminate ROS (Dhir et al., 2012). The decrease of proline contents in L3 treatments might be explained by the fact that the physiological functions and metabolisms of plants were seriously damaged at high concentration of Pb–Zn toxicity (Chen et al., 2003). When the equilibrium between ROS generation and detoxification was disrupted by abiotic stresses, the induction of antioxidative enzyme defense activities played a crucial role in HM tolerance in plants (Ashraf, 2009). SOD was the first defense line against oxidative stresses, and superoxide radicals (O_2_^−^) were scavenged by SOD. CAT was recognized as the most important enzyme for scavenging H_2_O_2_ produced in plant cells and was primarily associated with the maintenance steady of cellular. POD enzyme can detoxify H_2_O_2_ and thereby was conducive to maintaining the integrity of cellular membranes (Liu et al., 2017). In this study, SOD, POD, and CAT activity in *M. azedarach* and *L. lucidum* initially increased and then declined under Pb–Zn stress (**Figure 8**). The same patterns were found in *Iris halophila* exposed to Pb mine tailing treatments (Han et al., 2016). The SOD, POD, and CAT activities were higher in the L1 and L2 treatments than those in the C, indicating that the antioxidative system can effectively mitigate oxidative damage (Nie et al., 2016). The reduction of antioxidative enzyme activity under the highest Pb–Zn tailing treatments might be ascribed to the fact that the gene expression of SOD, POD and CAT enzymes was impacted by strong Pb–Zn stress (Hu et al., 2015). Another account for the decrease in SOD, POD, and CAT activity was that these variables were exhausted to alleviate the detrimental effects of ROS under higher Pb–Zn stress (Nie et al., 2016).

Under Pb–Zn stress, *L. lucidum* had a lower level of lipid peroxidation and higher activities of the antioxidative enzyme when compared to *M. azedarach*. As the tolerance to HMs was linked with the low level of lipid peroxidation and high activities of antioxidative enzymes (Ma et al., 2001; Sharma and Dietz, 2009), *L. lucidum* likely possessed a higher tolerability to HMs than *M. azedarach*. Additionally, less influence occurred on PSII and PSI in *L. lucidum* than in *M. azedarach* (**Figure 4**), which was probably attributed to the lower MDA content and higher antioxidative enzyme activities in *L. lucidum* (Iqbal et al., 2019). It should be noted that the TFs for Pb and Zn in stems and leaves were lower in *L. lucidum* than in *M. azedarach*, and more Pb and Zn were retained in *L. lucidum* roots, indicating that *L. lucidum* has more excellent metal exclusion strategy under Pb–Zn mine tailing treatments.

## Conclusion

The current study showed that Pb–Zn mine tailing had a crucial negative influence on two tested plants’ development and biomass production by inhibiting the chlorophyll synthesis and photosynthetic metabolism. The reduction of net photosynthetic rates in *M. azedarach* and *L. lucidum* due to HM stress was mainly caused by their biochemical limitation, including decreases of V_cmax_ and J_max_. Pb–Zn stress damaged multiple locations along the photosynthetic electron transport chain. Specifically, it impaired the OEC (only in *M. azedarach*) and blocked electron ﬂow of acceptor side of PSII and disturbed the PSI oxidation and reduction in both tested trees. Moreover, the increase of ROS content in both plant species was directly related to the obstruction of the electron transfer. Meanwhile, *M. azedarach* and *L. lucidum* could maintain high levels of SOD, POD, CAT, and proline contents to effectively relieve oxidative stress. The more tolerance of *L. lucidum* might be attributed to these facts: (1) higher RGR; (2) more accumulation of Pb–Zn in roots; (3) a less extent effect occurred on PSII and PSI activity; and (4) lower ROS and MDA content and higher antioxidative enzymes activities.

## Data Availability Statement

All datasets presented in this study are included in the article.

## Author Contributions

Idea and study designed: FZ. Performed the experiments: ML and HX. Wrote the paper: XH. Helped revise original paper: XC, ZH, and GW.

## Funding

This work was financially supported by the Key Research and Development Project of Hunan Province (2017NK2171), the “948” introduction project of The State Bureaucracy of Forestry (2014-4-62), the Hunan Provincial Innovation Foundation For Postgraduate (CX2018B434), the Scientific Innovation Fund for Post-graduates of Central South University of Forestry and Technology (20181007), and the Scientific Innovation Fund for Post-graduates of Central South University of Forestry and Technology (CX20192062).

## Conflict of Interest

The authors declare that the research was conducted in the absence of any commercial or financial relationships that could be construed as a potential conflict of interest.
